# Supplementary material for: Potential Role of Macrophage Polarization in the Progression of Hunner-Type Interstitial Cystitis
Source: Int J Mol Sci. 2024 Jan 8;25(2):778. doi: 10.3390/ijms25020778 (PMC10815545; doi:10.3390/ijms25020778)
Supplement: Supplementary file 1 [file ijms-25-00778-s001.zip › Supplementary figures.pdf]

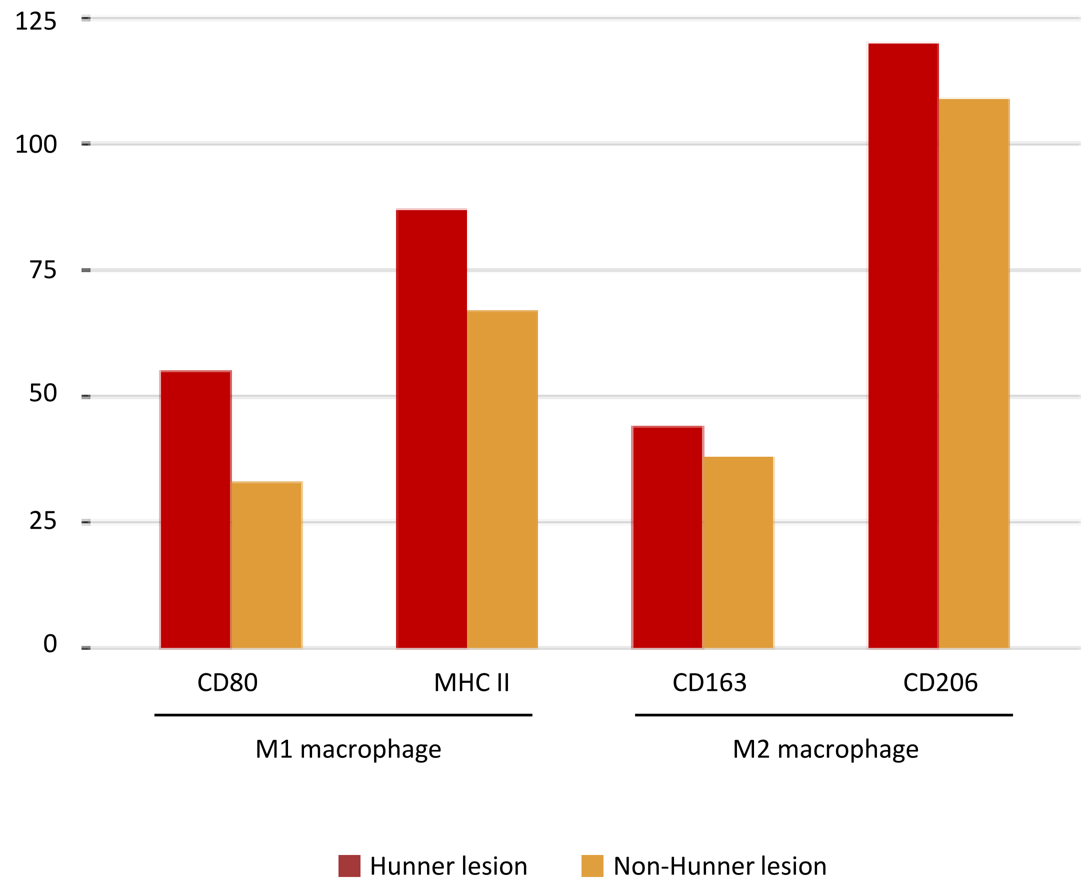

Supplementary Figure S1. Cell count (Numbers per high-power field) of M1 macrophages (CD80 and MHC II) and M2 macrophages (CD163 and CD206) in Hunner and non-hunner lesions.

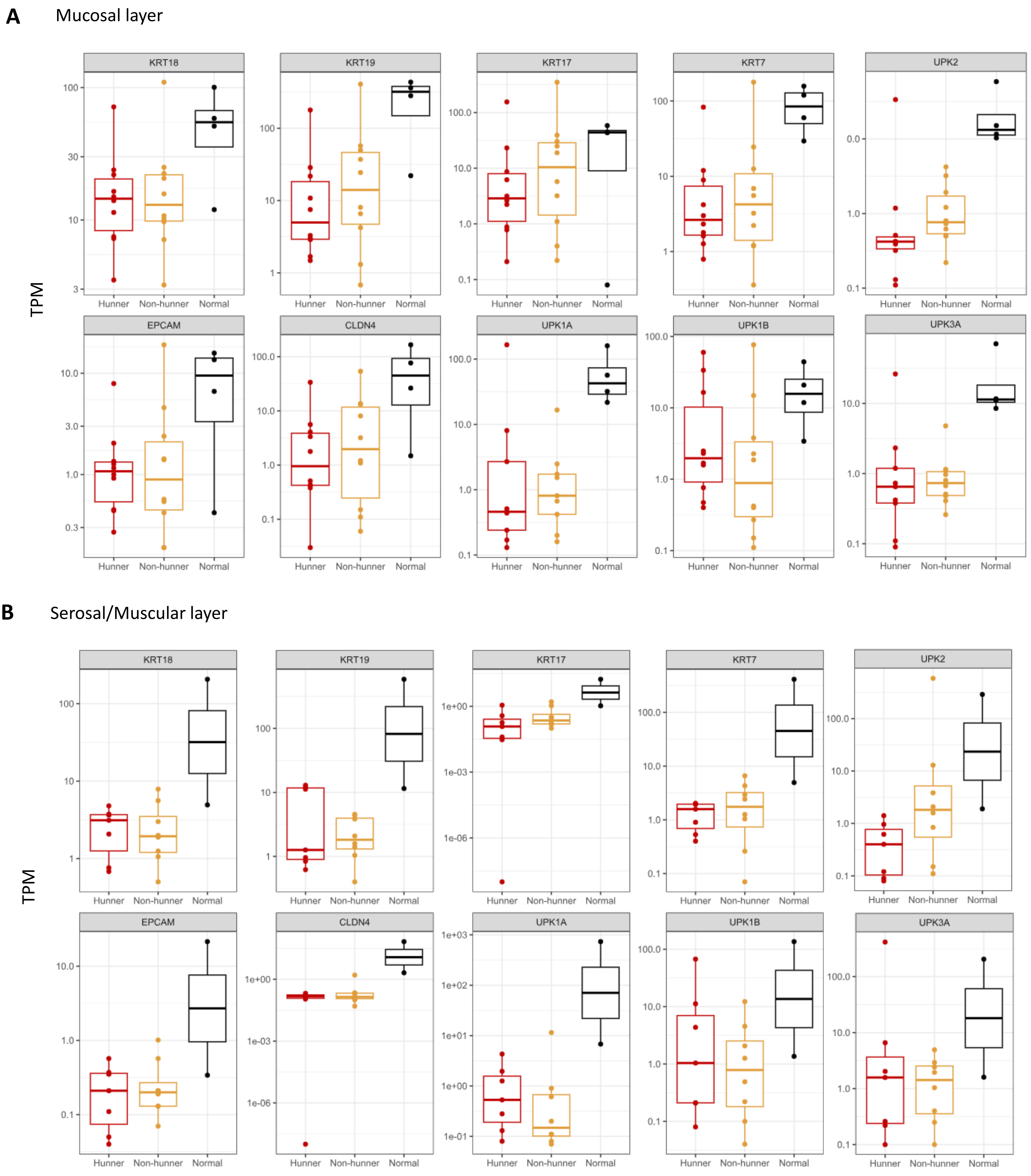

Supplementary Figure S2. Expression of epithelial marker genes in mucosal layer (A) and serosal/muscular layer (B).
